# Phase-contrast magnetic resonance imaging of intracranial and extracranial blood flow in carotid near-occlusion

**DOI:** 10.1007/s00234-024-03309-y

**Published:** 2024-02-24

**Authors:** Madelene Holmgren, Alexander Henze, Anders Wåhlin, Anders Eklund, Allan J. Fox, Elias Johansson

**Affiliations:** 1 Department of Clinical Sciences, Neurosciences, Umeå University, Umeå, Sweden; 2 Department of Radiation Sciences, Biomedical Engineering, Umeå University, Umeå, Sweden; 3 Umeå Center for Functional Brain Imaging, Umeå University, Umeå, Sweden; 4 Department of Applied Physics and Electronics, Umeå University, Umeå, Sweden; 5 Sunnybrook Health Science Center, University of Toronto, Toronto, Canada; 6 Wallenberg Center for Molecular Medicine, Umeå University, Umeå, Sweden; 7 Neuroscience and Physiology, Gothenburg University, Göteborg, Sweden

**Keywords:** Carotid stenosis, Carotid near-occlusion, Intracerebral flow, Collaterals, Phase-contrast MRI, CT angiography

## Abstract

**Purpose:**

Compare extracranial internal carotid artery flow rates and intracranial collateral use between conventional ≥ 50% carotid stenosis and carotid near-occlusion, and between symptomatic and asymptomatic carotid near-occlusion.

**Methods:**

We included patients with ≥ 50% carotid stenosis. Degree of stenosis was diagnosed on CTA. Mean blood flow rates were assessed with four-dimensional phase-contrast MRI.

**Results:**

We included 110 patients of which 83% were symptomatic, and 38% had near-occlusion. Near-occlusions had lower mean internal carotid artery flow (70 ml/min) than conventional ≥ 50% stenoses (203 ml/min, P < .001). Definite use of ≥ 1 collateral was found in 83% (35/42) of near-occlusions and 10% (7/68) of conventional stenoses (P < .001). However, there were no differences in total cerebral blood flow (514 ml/min vs. 519 ml/min, P = .78) or ipsilateral hemispheric blood flow (234 vs. 227 ml/min, P = .52), between near-occlusions and conventional ≥ 50% stenoses, based on phase-contrast MRI flow rates. There were no differences in total cerebral or hemispheric blood flow, or collateral use, between symptomatic and asymptomatic near-occlusions.

**Conclusion:**

Near-occlusions have lower internal carotid artery flow rates and more collateral use, but similar total cerebral blood flow and hemispheric blood flow, compared to conventional ≥ 50% carotid stenosis.

**Supplementary Information:**

The online version contains supplementary material available at 10.1007/s00234-024-03309-y.

## Introduction

**Fig. 1:**
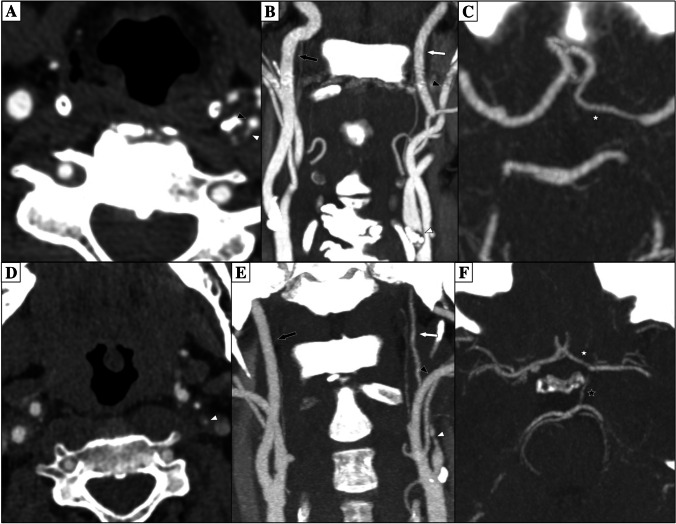
CTA of two examples of left-sided near-occlusion. (**A**-**C**) Near-occlusion without full collapse. (**A**-**B**) After a severe stenosis, the distal ICA is normal-appearing but smaller than the contralateral ICA and approaching the size of ipsilateral external carotid artery. Parts of the distal left ICA not in the plane. (**C**) Left A1 smaller than right, Pcom not seen. On PC-MRI, ICA flows were 312 ml/min right and 72 ml/min left; A1 flows were 185 ml/min right and -42 ml/min (reversed) left; Pcom and OA not seen bilaterally. (**D**-**F**) Near-occlusion with full collapse. (**D**-**E**) After a severe stenosis, the distal left ICA is threadlike, clearly smaller than the contralateral ICA and ipsilateral external carotid artery. (**F**) Virtually similar A1s and left Pcom seen. On PC-MRI, ICA flows were 264 ml/min right and seen as weak antegrade signal left (not assessable by the algorithm, approximated to 10 ml/min); A1 flows were 198 ml/min right and -61 ml/min (reversed) left; Pcom left -52 ml/min (towards the MCA), right not seen; OA right antegrade, left not seen. In all figures: White arrowhead is stenosis; White arrow is ipsilateral distal ICA; Black arrowhead is ipsilateral external carotid artery; Black arrow is contralateral ICA; White star is ipsilateral A1; Black star is probable ipsilateral Pcom;

Carotid near-occlusion is a severe carotid stenosis that causes distal artery size reduction (“collapse”) [1, 2]. The size reduction is often moderate, and the distal artery is still normal-appearing (near-occlusion without full collapse, Fig. 1A-C), but can also be more severe (near-occlusion with full collapse, Fig. 1D-F) [1, 2]. In contrast, most ≥ 50% carotid stenoses do not cause distal artery size reduction (conventional stenosis) [1, 2]. Differentiating between near-occlusion and conventional stenosis is relevant because their recommended treatment management differs [3].

Phase-contrast MRI (PC-MRI) can assess arterial flow rates (in milliliters/minute) and flow directions and, thereby, collateral use of the larger arteries in Circle of Willis. Such flow rates are of arterial perfusion, not of brain regional perfusion. Phase-contrast MRI studies have virtually unanimously found an association between an increasing degree of carotid stenosis and a reduction in flow rates through the internal carotid artery (ICA) and, when assessed, increased collateral use [4–15]. However, until recently, no PC-MRI studies assessed near-occlusions separately. In two recent exceptions, flow rate reduction in ICA seemed to only occur in near-occlusion [6, 15]. In one of these studies, a strong correlation between ICA flow rate and distal ICA size was suggested, i.e. the hallmark of near-occlusion [6]. However, this study was small (n = 29) and did not assess collateral use [6]. The other study was limited to diagnostic analyses and only presented mean flow rates in Circle of Willis artery segments, not how often collaterals were used [15]. Thus, it remains unclear if flow rate reduction in ICA and increased collateral recruitment of major intracranial arteries are valid for severe carotid stenosis in general (severe conventional stenosis and near-occlusion) or are specific for near-occlusion.

In addition, the mechanism of stroke in near-occlusion is unclear and could be hypoperfusion, from insufficient blood flow to the brain [2]. If so, cerebral blood flow, as assessed from summed PC-MRI flow rates of supplying arteries, should reasonably be worse in symptomatic near-occlusion than asymptomatic near-occlusion, but this has never been assessed with PC-MRI.

The aim of this study was to compare extracranial ICA flow and intracranial collateral use between conventional ≥ 50% stenosis and near-occlusion, and between symptomatic and asymptomatic near-occlusion.

## Methods

### Study cohort

The study setting was a single tertiary stroke center (Umeå Stroke center), serving as the only carotid stenosis evaluation center for 11 referring hospitals with a population of approximately 900 000. Between June 2018 and November 2021, we prospectively asked patients evaluated for possible carotid endarterectomy or stenting for symptomatic ≥ 50% carotid stenosis to participate in the study. Symptomatic was defined as a recent (< 6 months) ipsilateral ischemic stroke, TIA, or monocular blindness (retinal events: amaurosis fugax when transient, retinal artery occlusion when non-transient). While a consecutive sample was sought for symptomatic patients, this was occasionally logistically difficult given short evaluations before surgery/stenting (never delayed for the study MRI) and staff issues (turnover, vacations, and clinical work during Covid19 pandemic). Persons with asymptomatic carotid stenosis did not undergo preoperative evaluations at our center. Included persons with asymptomatic ≥ 50% stenoses were either evaluated for symptomatic stenosis, but had none, or were known from previous studies and summoned specifically for study participation. A diagnostic analysis of this material has previously been presented [15].

For all cases, the inclusion criterium was ≥ 50% stenosis on at least one cervical side on CTA. Exclusion criteria were CTA or MRI contraindications, unable to walk to and lay in the MRI camera, and unable to provide informed consent. The study was approved by the Regional ethics board in Umeå, Sweden.

### CTA assessment

CTAs were assessed by three expert observers blinded to each other and to MRI findings. EJ (10 years’ experience), assessed all exams and collected CTA diameters, and all cases with any suspicion of near-occlusion were always assessed by either AJF (> 40 years’ experience) or AH (5 years’ experience). Disagreements about near-occlusion diagnosis were resolved by consensus discussion, and disagreements about above or below 50% stenosis were resolved by deferring to carotid ultrasound findings (n = 2). Cases with small distal ICA of unclear cause were excluded from the main analyses.

Degree of stenosis for cases with conventional carotid stenosis was assessed using NASCET methodology [16]. Carotid near-occlusion diagnosis by feature interpretation [17], and definition of full collapse is described elsewhere [18]. Occlusion was diagnosed when no contrast was seen beyond the stenosis. Anatomical variants of Circle of Willis as an explanation for small distal ICA were considered a subset of conventional stenosis [19]. In severe stenosis cases, CTA was limited by partial volume effects in accurately assessing artery diameters. In such cases, distal ICA and stenosis lumen diameters were approximated to 0.5 and 0.2 mm when visible or not visible contrast. Figure processing was done using RadiAnt DICOM Viewer (radiantviewer.com).

### Phase-contrast MRI

The four-dimensional PC-MRI scans were performed on a 3 T scanner (GE Discovery MR 750, Milwaukee, WI, USA) with a 32-channel head coil. The scan time sequence was ≈9 min and provided a full brain coverage of velocities in three spatial directions [20, 21]. Imaging parameters were: TR/TE 6.5/2.7 ms, velocity encoding 110 cm/s (standard artery flow Venc), flip angle 8°, 16,000 radial projections, acquisition resolution 300 × 300 × 300, reconstructed resolution 320 × 320 × 320, imaging volume 220 × 220 × 220 mm3 isotropic voxel size 0.69 mm. The data was reconstructed into an angiographic complex difference image for vessel identification and segmentation, and time-resolved three-directional velocities with 20 frames per cardiac cycle by pulse oximeter trigger. Mean blood flows, which were obtained by averaging the pulsatile waveform in each artery. No contrast agent was used in the MRI scans.

**Fig. 2:**
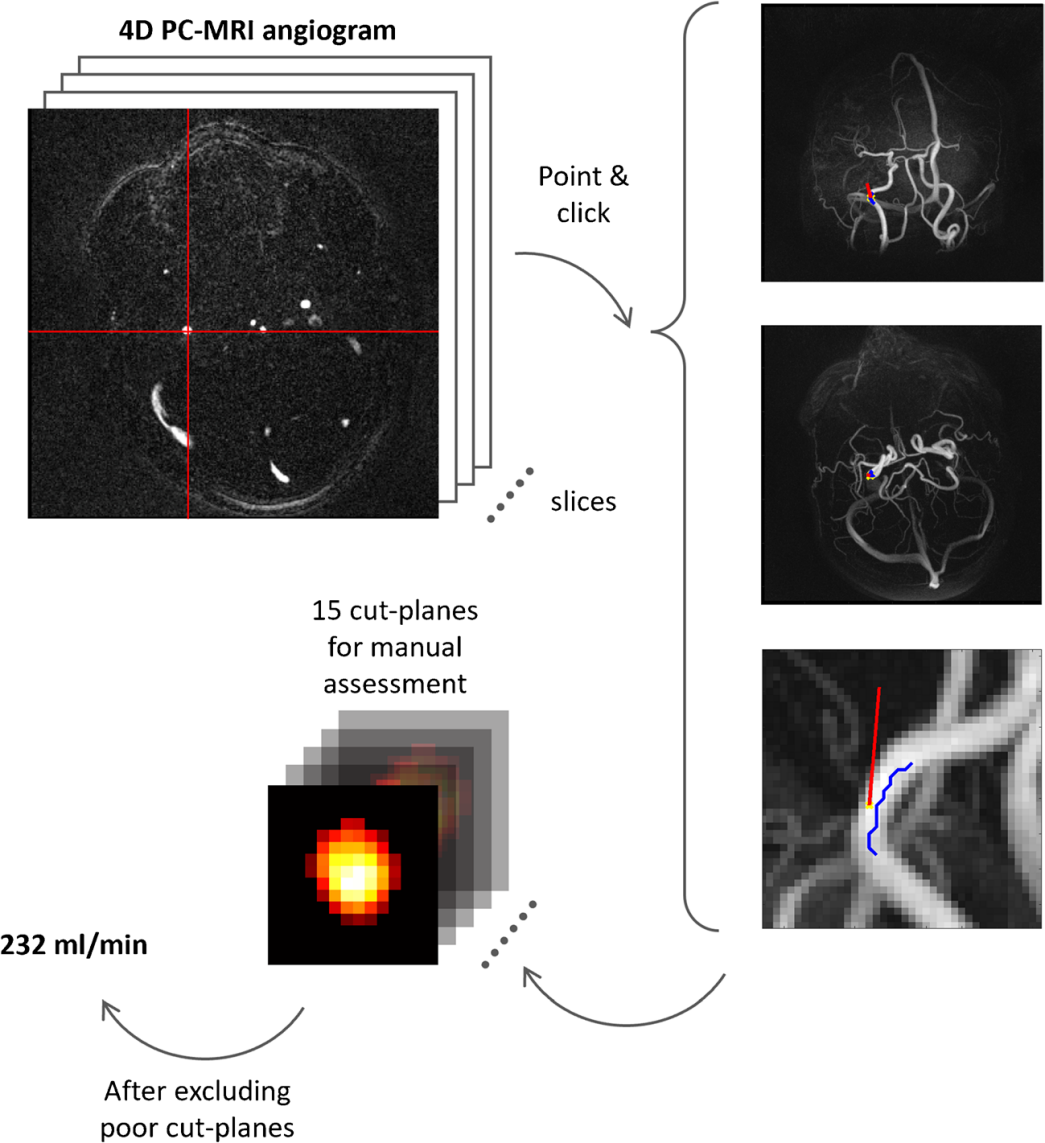
Workflow of MRI analysis. The user selected an artery from the axial view in the four-dimensional PC-MRI volume angiogram (internal carotid artery in this example, red cursor). Visualizations in three anatomical directions were used to inspect the chosen arterial segment (blue line) and flow direction (red line). Cross-sectional angiographic planes were aligned perpendicular to the flow direction in 15 consecutive planes, followed by automatic segmentation and visual inspection of the arterial lumen in each plane. After the user choose which cut-planes that should be discarded (if any), the flow rate in the chosen artery was calculated by the software and presented to the user

Blood flow rates were quantified from the MRI data using a previously developed post-processing algorithms using MATLAB (R2020b, The MathWorks Inc., Natick, MA, USA) [22, 23] The arterial segment of interest was manually selected from the angiogram (Fig. 2). Arterial lumen was segmented from perpendicular cross-sectional planes, using a maximum image intensity threshold locally in each plane. The flow rate in each plane was calculated as the average velocity multiplied by the cross-sectional area. The threshold parameter of initially 16% was iteratively increased in steps of 1%, up to maximum 24%, if the coefficient of variation between the flow rates from the cut-planes was > 15% (a step added for this study). This was rarely necessary for the larger arteries (ICA, BA, M1). All final cut-planes were inspected, and planes with a nearby artery still persistent in the image were excluded (Supplement Figure S1). The flow in an artery was calculated by averaging the included cut-planes.

MH (6 years’ experience) assessed all MRI exams and discussed difficult cases with EJ. The flow rates were assessed on both the ipsi- and contralateral sides in the ICAs (just below the skull base), ophthalmic arteries (OA), middle cerebral arteries at M1 level, anterior cerebral arteries at A1 and A2 level, posterior cerebral arteries at P1 and P2 level, posterior communicating arteries (Pcom), and the basilar artery (BA). Anterior communicating artery was too short to measure. Total cerebral blood flow (CBF_tot_) was obtained from the summed flow of the ipsi- and contralateral ICAs, and the BA. The summed flow of A2, M1, and P2 gave the hemispheric flow (Hemi_tot_) for each side separately. Some A2s were too close to one another and, therefore, not separable (Supplement Figure S1). In such cases, A2s were assessed with a previously published approach, using the same angiographic images, but applying an image intensity threshold based on the whole image volume, without using perpendicular planes.

Flow directions through arterial segments visible in the angiograms, but not interpretable by the software, were determined from velocity images. Based on the strength of the angiogram signal, the flow rates were approximated to 20 ml/min (weak signal) or 10 ml/min (very weak signal). These assigned values were based on that artery segments with the lowest flow that still were interpretable by the software had flow rate slightly above 20 ml/min. Internal carotid arteries with no visible flow on the angiogram, but visible on CTA, were set to antegrade 5 ml/min as flow rate and flow direction could be assumed. Otherwise, arterial segments not visible on the angiogram were set to 0 ml/min.

### Collateral flow

Collateral arteries analyzed were the ipsilateral OA, Pcom, and A1. Antegrade flow directions were defined as ICA to eye for OA, ICA to A2 for A1, and ICA to P1 for Pcom. Collateral flow in OA and Pcom were divided into antegrade, not seen, and reversed. To include the gradual recruitment of A1 collaterals suggested by Zarrinkoob et al. [14], A1 flow rates were arbitrarily divided into normal (> 33% of the summed bilateral A1 flow), reduced (1–33%), not seen, and reversed. The number of collaterals was defined as either possible (reduced, not seen, or reversed) or definite (only reversed).

### Statistics

The main analysis was based on conventional ≥ 50% stenosis, and symptomatic and asymptomatic near-occlusion. We compared near-occlusions to conventional ≥ 50% stenoses, and symptomatic to asymptomatic near-occlusions. Results for blood flow rates were expressed in mean and standard deviations (SD) and blood flow ratios in the median and interquartile ranges (IQR). Where appropriate, we used independent sample T-test, two-sided χ^2^ test, Mann–Whitney U-test, Pearson’s linear correlation coefficient, and linear regression. The significance level was set at P < 0.05. Statistics were performed in SPSS 28.0 (IBM).

## Results

**Fig. 3:**
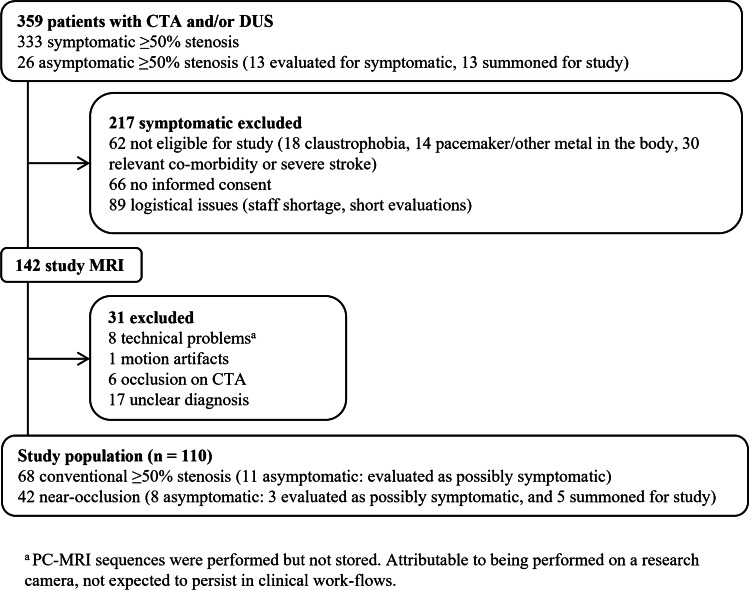
Study flow chart. CTA, computed tomography; DUS, digital ultrasound; MRI, magnetic resonance imaging

One hundred and ten cases were included in the main analysis, where 68 had conventional ≥ 50% stenosis (11 asymptomatic) and 42 were near-occlusions (8 asymptomatic). Mean age was 73 years, and 32% were women. See Fig. 3 for study flow chart and Supplement Table S1 for Baseline characteristics. Blood flow rates for the stenosis groups are shown in Table 1, and flow rates for the 17 cases with unclear diagnosis in Supplement Table S2.

**Table 1:** Comparison of blood flow and blood flow ratios

	Conventional ≥ 50% stenosis (n = 68)	Near-occlusion (n = 42)	p	Symptomatic near-occlusion (n = 34)	Asymptomatic near-occlusion (n = 8)	p
Blood flow, mean (SD) ml/min						
CBF_tot_	519 (88)	514 (88)	.78	516 (93)	504 (70)	.74
Hemi_tot_-ipsi^b^	227 (55)	234 (51)	.52	238 (52)	217 (49)	.34
Hemi_tot_-contra^b^	240 (64)	250 (60)	.45	247 (63)	263 (41)	.54
ICA-ipsi	203 (73)	70 (45)	< .001	77 (44)	40 (34)	.03
ICA-contra^c^	209 (69)	267 (85)	< .001	263 (92)	283 (48)	.45
M1-ipsi	121 (28)	104 (38)	.01	108 (36)	97 (36)	.44
M1-contra	125 (37)	128 (44)	.72	127 (47)	133 (21)	.71
A1-ipsi	78 (59)	-10 (43)	< .001	-5 (41)	-34 (46)	.08
A1-contra	64 (64)	133 (62)	< .001	126 (64)	164 (41)	.12
A2-ipsi^b^	54 (19)	57 (23)	.47	57 (24)	56 (21)	.89
A2-contra^b^	59 (20)	55 (19)	.36	53 (20)	65 (12)	.14
BA	122 (48)	183 (61)	< .001	183 (63)	181 (58)	.94
P1-ipsi	50 (34)	100 (40)	< .001	103 (39)	89 (44)	.39
P1-contra	47 (31)	67 (53)	.02	67 (58)	68 (28)	.95
P2-ipsi	54 (22)	71 (28)	< .001	73 (27)	66 (30)	.53
P2-contra	58 (20)	68 (31)	.03	68 (33)	65 (14)	.81
Pcom-ipsi	7 (22)	-28 (33)	< .001	-30 (34)	-26 (31)	.81
Pcom-contra	12 (30)	8 (30)	.50	10 (32)	2 (23)	.55
Blood flow ratios, median (IQR)						
Hemi_tot_-ipsi/contra^b^	94 (87 − 105)	92 (84 − 100)	.41	93 (85 − 100)	85 (67 − 91)	.06
ICA-ipsi/ICA_sum_	46 (43 − 54)	20 (13 − 29)	< .001	22 (15 − 32)	13 (2 − 16)	.01
ICA-ipsi/CBF_tot_	36 (32 − 42)	13 (8 − 18)	< .001	15 (9 − 19)	8 (1 − 11)	.02
M1-ipsi/contra^d^	95 (86 − 108)	82 (70 − 95)	< .001	82 (72 − 96)	76 (67 − 86)	.39
A1-ipsi/A1_sum_	45 (31 − 62)	0 (-41 − 10)	< .001	0 (-28 − 16)	-22 (-56 − 0)	.13

Negative numbers indicate reversed flow. CBF_tot_ = ICA-ipsi + ICA-contra + BA; Hemi_tot_ = M1 + A2 + P2; ICA_sum_ = ICA-ipsi + ICA-contra; A1_sum_ = A1-ipsi + A1-contra

^a^ Two-sided independent sample t-test for flow ratios. Mann–Whitney U-test for flow ratios

^b^ 6 missing MRI due to the A2s being too close together to assess individually (2 near-occlusions)

^c^ 6 missing due to contralateral occlusion (1 near-occlusion)

^d^ 1 missing due to bilateral MCA-occlusion on both CTA and MRI (near-occlusion)

### Near-occlusion compared to conventional ≥ 50% stenosis

**Fig. 4:**
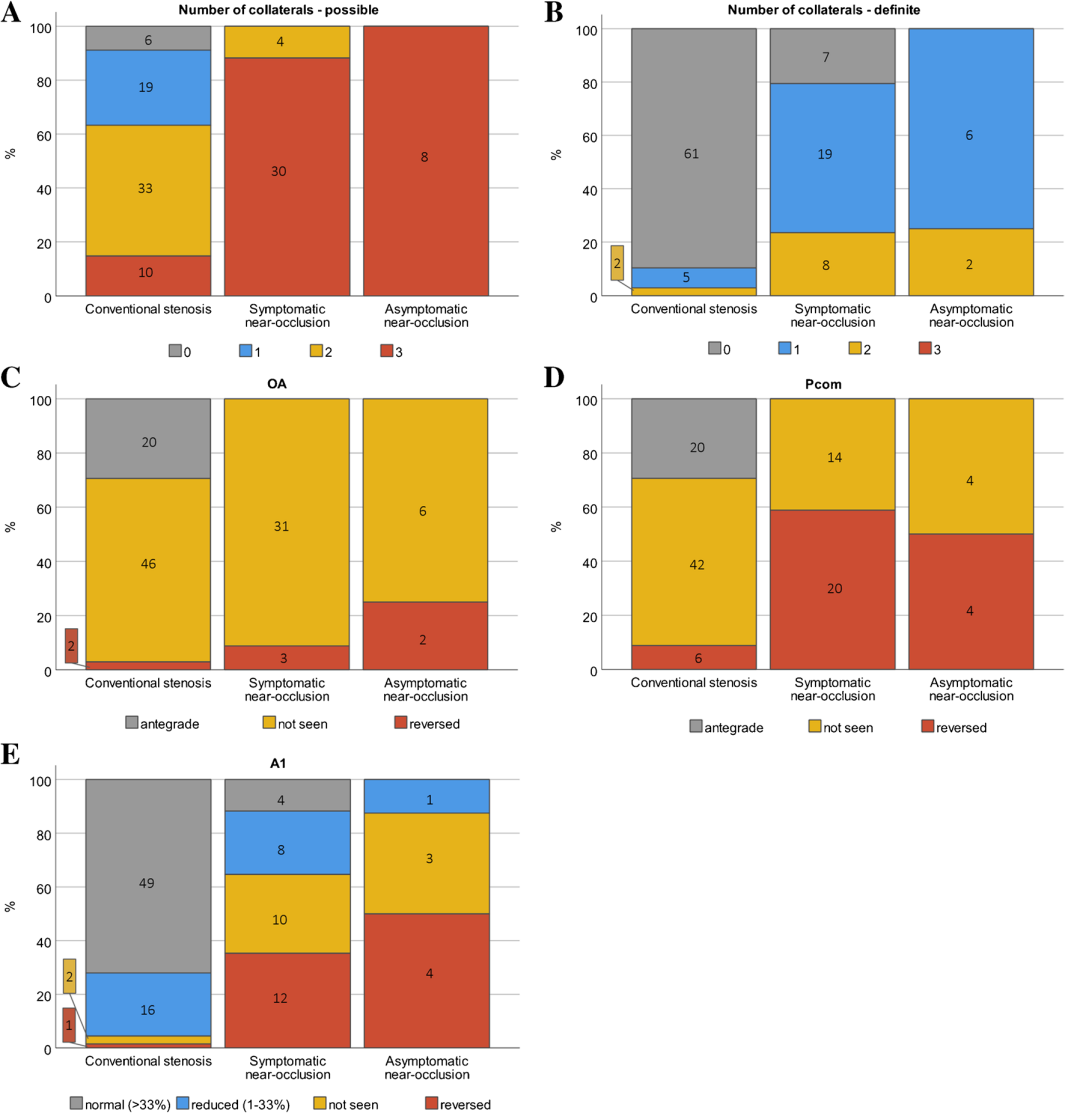
Collateral use. (**A**-**B**) Total number of recruited ipsilateral collaterals, when defined as possible (reduced, not seen, reversed) or definite (only reversed). There are no cases with definite use of all three collaterals. (**C**-**E**) Collateral use of the ipsilateral side of ophthalmic artery (OA), posterior communicating artery (Pcom), and anterior communication artery (A1), respectively. Near-occlusion was different compared to conventional stenosis in all cases (p < .001, Mann–Whitney), but symptomatic near-occlusions were not different from asymptomatic near-occlusions (A p = .63, B p = .49, C p = .50, D p = .72 and E p = .28)

Near-occlusions had significantly different flow rates and flow rate ratios in various artery segments, consistent with lower ipsilateral ICA flow rates and increased collateral use, primarily manifested by reversed flow rates in the ipsilateral A1 and Pcom, and an increased flow rate in the contralateral A1 (Table 1). The flow differences remained when comparing near-occlusions and conventional ≥ 70% stenoses (Supplement Table S3). Differences were also confirmed in ordinal analysis by a higher number of recruited collaterals and more collateral use in near-occlusions (Fig. 4). Definite use of ≥ 1 collateral was found in 83% (35/42) of near-occlusions and 10% (7/68) of conventional stenoses, P < 0.001). Consistent with the pattern of compensation through collateral use, there were no differences in CBF_tot_ or ipsi- and contralateral Hemi_tot_. In addition, near-occlusions had lower ipsilateral M1 flow rates and higher ipsilateral P2 flow rates, but no difference in A2 flow rates, indicating ipsilateral leptomeningeal collateral flow from the P2 to M1 vascular territories. However, higher flow rates in the contralateral P1 and P2 were inconsistent with a pattern of ipsilateral compensation.

### Symptomatic compared to asymptomatic near-occlusion

Symptomatic near-occlusions did not have significantly different flow rates than asymptomatic near-occlusions in any arterial segment except in the ipsilateral ICA, nor was the CBF_tot_ or Hemi_tot_ different (Table 1). The ipsilateral ICA flow rate ratios were lower in asymptomatic near-occlusions than in symptomatic (Table 1). The collateral use and the number of recruited collaterals were not significantly different between symptomatic and asymptomatic near-occlusion (Fig. 4).

CTA data indicated that the included asymptomatic near-occlusions were more severe than the symptomatic, with smaller mean distal ICA diameter and more often full collapse (Supplement Table S1). Near-occlusions with full collapse had lower ipsilateral ICA flow rates than near-occlusions without full collapse (Supplemental Table S4). After adjusting for full collapse status in linear regression models, there were no changes with respect to differences in any flow rates between symptomatic and asymptomatic near-occlusions (data not shown).

### Relationships between ICA flow rate, area, and stenosis degree

**Fig. 5:**
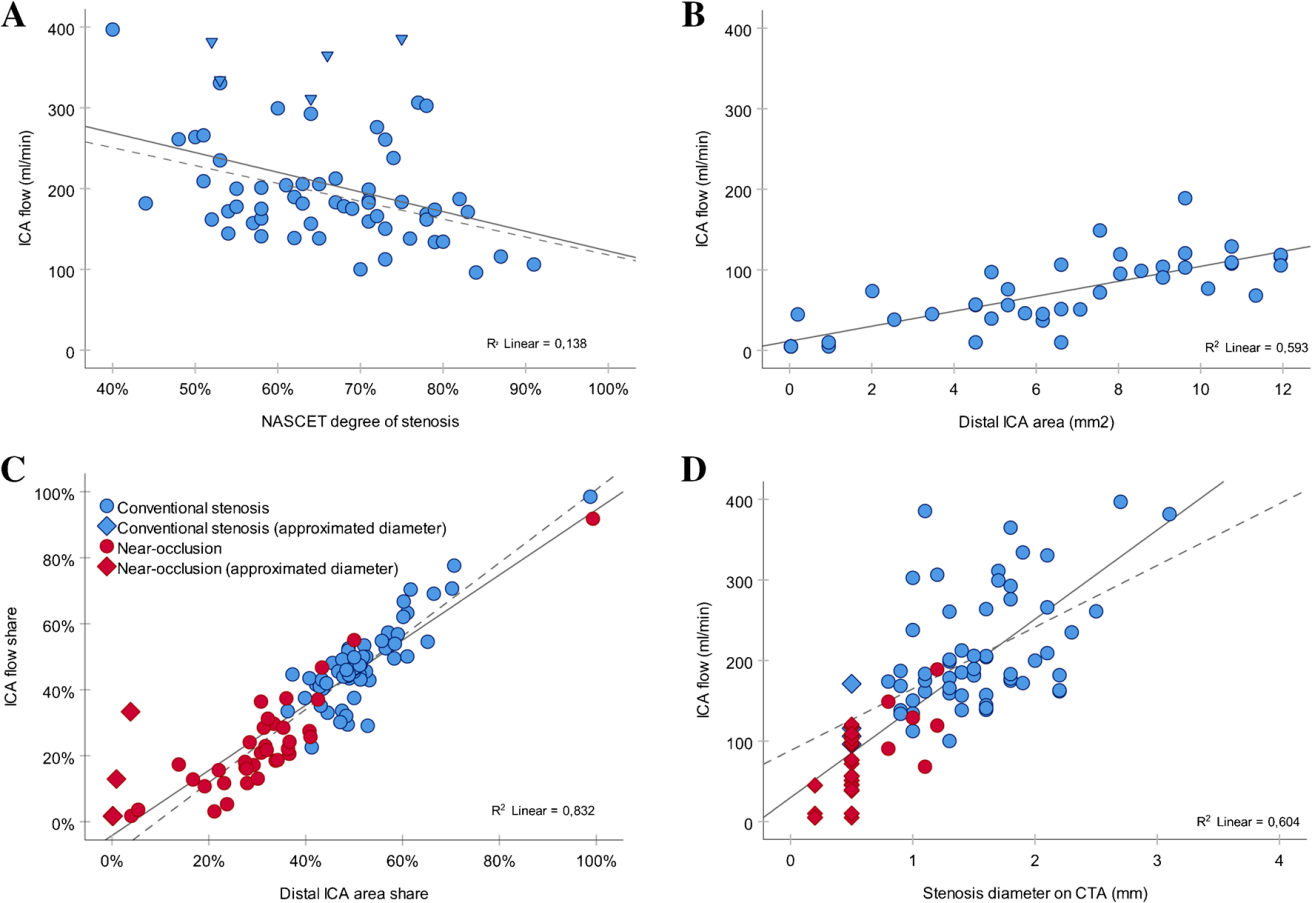
Relationship between ICA flow and diameters. (**A**-**B**) Ipsilateral ICA flow rate versus (**A**) NASCET degree of stenosis in conventional ≥ 50% stenoses with dashed regression line when excluding cases with contralateral ICA occlusion (triangles), and (**B**) distal ICA area in near-occlusions. (**C**-**D**) Relative ipsilateral ICA flow rate (share) versus (**C**) relative distal ipsilateral ICA area, and (**D**) ipsilateral ICA flow rate versus stenosis diameter on CTA. Note the many cases with approximated diameters of 0.2- and 0.5-mm (squares), with dashed regression lines when excluding these diameters

The ipsilateral ICA flow rates were decreasing with an increasing degree of stenosis for conventional stenoses (r = -0.37, P = 0.003, Fig. 5A). This association remained when excluding five cases with contralateral occlusion (r = -0.41, P = 0.001). Flow rates in near-occlusions were decreasing with a decreased distal ICA area (r = 0.78, P < 0.001, Fig. 5B). A strong positive relationship between the ICA flow rates and distal ICA area was also illustrated in relative terms for both conventional stenoses and near-occlusions (r = 0.91, P < 0.001, Fig. 5C). Excluding cases with approximated distal diameters, the correlation was r = 0.93 (P < 0.001). Ipsilateral ICA flow rates, additionally, had a positive relation to stenosis diameters, although most near-occlusions consisted of approximated diameters (r = 0.78, P < 0.001, Fig. 5D). Excluding approximated stenosis diameters, the correlation decreased but was still significant (r = 0.50, P < 0.001).

## Discussion

The main findings were that carotid near-occlusions had lower ipsilateral ICA flow rates and more intracranial collateral use compared to conventional ≥ 50% stenoses, while there were no relevant differences between symptomatic and asymptomatic near-occlusions. Almost all near-occlusions and only a few conventional stenoses had definite collateral use.

Our study is the first to clearly describe the intracranial blood flow distribution (in ml/min) in patients with carotid near-occlusion, with findings consistent with understandings from conventional angiography [1, 2] and distal ICA velocity assessments in carotid ultrasound [24, 25]. It is also only the third PC-MRI study investigating near-occlusion separately (of which one used the same materials for a diagnostic analysis) [6, 15], and it contains a larger study population than previous studies investigating the relation between carotid stenosis and ICA blood flow [4–14]. Further comparisons of pathophysiology markers are warranted, such as comparing stenosis velocity and distal ICA velocity on ultrasound and PC-MRI.

We found a reduced ipsilateral ICA flow, and an extensive ipsilateral collateral use, with increasing stenosis severity regarding both conventional stenosis and near-occlusion, in line with a previous four-dimensional PC-MRI study [14]. Our correlation between the ICA flow rate and the degree of stenosis among conventional stenoses, also after excluding cases with a contralateral occlusion, contradicted the previous proof-of-concept study [6]. These findings suggest that a flow rate reduction through the ipsilateral ICA and increased collateral use exist in both conventional stenosis and near-occlusion, but are markedly more common in near-occlusion. However, as previously seen [6], there was a very strong correlation between relative ICA flow and area in the whole range of stenoses. This correlation makes it unlikely that stenoses can cause a clear reduction of flow rates without also affecting distal ICA size. Therefore, it was not surprising that ICA flow rate was a good prediction for separation conventional stenosis and near-occlusion in diagnostic studies, albeit it turned out that relative flow was slightly better [6, 15]. This is clinically useful since near-occlusion is often overlooked in CTA [17]. As near-occlusions often have high flow velocities in the stenosis on carotid ultrasound, they are not easily distinguishable from conventional stenoses [26], why methods for clinical use in addition to ultrasound are warranted and ultrasound is not very useful as reference method in near-occlusion studies [15].

We had a difference in the severity of stenosis (by distal ICA diameter) between symptomatic and asymptomatic near-occlusions and a high share of near-occlusion among asymptomatic cases. This was likely an effect of study selection (summoning cases), as a recent study without such selection found no such differences in angiographic appearance [27]. We found similar cerebral and hemispheric blood flow rates in symptomatic and asymptomatic near-occlusions, which remained after adjusting for collapse status. If the mechanism of stroke in symptomatic near-occlusion was cerebral hypoperfusion, a lower hemisphere blood flow should have been expected in symptomatic compared to asymptomatic near-occlusions. As the tendency was towards the opposite in our data, the neutral result was unlikely caused by a too small sample. Indeed, the side-to-side ratio of hemispheric blood flow was almost statistically significantly higher in symptomatic than asymptomatic near-occlusions (P = 0.06). Therefore, our results cannot confirm that hypoperfusion is the primary mechanism for stroke in near-occlusions. As blood flow rates are not an established method to assess hypoperfusion mechanism, a stronger conclusion would be unwarranted. However, these findings are in line with other studies: A recent study of 645 participants with ≥ 50% carotid stenosis (25% near-occlusions) found no differences in angiographic appearance between symptomatic and asymptomatic near-occlusions [27]. Similarly, while a reduced cerebrovascular reserve has been reported for symptomatic near-occlusion, exhausted reserve (a hallmark of hypoperfusion mechanism) has never been shown to be common (especially not in cases with full collapse), but rather the opposite [27–30]. Further analyses on the mechanism of stroke in near-occlusion are warranted, such as border zone infarction pattern and microemboli assessment on transcranial ultrasound.

Near-occlusions with full collapse had lower ipsilateral ICA flow rates and a larger and reversed ipsilateral A1 flow rate ratio, indicating increased collateral use compared to near-occlusions without full collapse. As the sample was limited, there might be additional relevant differences in intracranial flow, but the results are in agreement with previous findings in other modalities [1, 2, 6, 27].

The analysis of cases with unclear causes of small distal ICA showed that flow rates shared similar recruitment patterns as between conventional stenoses to near-occlusions. These unclear cases would benefit the most from improved diagnostics, but a final diagnosis would require a post-operative CTA [31], which is not yet an established method and is therefore beyond the scope of this study.

The whole spectrum of stenosis severity is covered by a percent degree in the ECST or CCA methods, but not in the NASCET method. With NASCET, cases with distal artery size reduction caused by the stenosis are graded as near-occlusion, not with percent [1]. Our findings show that such ICA size reduction is virtually synonymous with a flow rate reduction in ICA and increased collateral use. These findings do not contradict, but nuance, the previous understanding of the association between the degree of stenosis, flow rates, and collateralization. It should also be highlighted that near-occlusion is not rare, but rather underdiagnosed with ultrasound [26, 32] and when CTA is graded in routine practice [17]. A recent systematic review showed that approximately 30% of symptomatic ≥ 50% carotid stenosis is near-occlusion [33]. International guidelines differ slightly in recommended management of symptomatic near-occlusion [3, 34, 35]. A difference in prognosis has also been suggested between near-occlusion with and without full collapse, but if and how this should affect management is unclear [18]. Further assessment of the role of PC-MRI in routine diagnostics and prognostics is warranted.

There were several limitations. We did not use post-operative PC-MRI and can therefore not prove causality between the stenosis and differences in flow rates and collateral use, such an analysis would have been of particular interest for conventional stenosis. We only measured resting-state blood flow rates, not cerebrovascular reserve – which should be studied. The sometime non-consecutive sample for symptomatic patients may have introduced a selection bias. PC-MRI findings in small intracerebral arteries can be unreliable, but all cases were examined with the same protocol – it is unlikely that our main findings are caused by artefacts.

## Conclusions

Carotid near-occlusions have lower ICA flow rates and more intracranial collateral use compared to conventional ≥ 50% stenoses. No difference in total cerebral or hemispheric blood flow, or collateral use, was found between symptomatic and asymptomatic near-occlusions. This suggests that the mechanism of stroke in symptomatic near-occlusion is not hypoperfusion, strengthening the likelihood of other possible mechanism, such as a thromboembolic mechanism. More analyses are needed to explore how PC-MRI and cerebral blood flow can be used as diagnostics markers to separate conventional stenosis and near-occlusion.

### Supplementary Information

Below is the link to the electronic supplementary material.

Supplementary file1 (DOCX 77.2 KB)

## Data Availability

Data is available for checking calculations or collaborations upon reasonable request to Corresponding author.
