# Supplementary material for: Phase-contrast magnetic resonance imaging of intracranial and extracranial blood flow in carotid near-occlusion
Source: Neuroradiology. 2024 Feb 24;66(4):589–99. doi: 10.1007/s00234-024-03309-y (PMC10937755; doi:10.1007/s00234-024-03309-y)
Supplement: Supplementary file 1 — Supplementary file1 (DOCX 77.2 KB) [file 234_2024_3309_MOESM1_ESM.docx]

**Intracranial and extracranial blood flow in carotid near-occlusion**

ONLINE SUPPLEMENTAL DATA

A

B

**Supplemental Figure S1.** Perpendicular angiographic cut-planes from the flow rate quantification algorithm. (A) Example of segmentation of the anterior cerebral artery (A1 segment), where the last cut-plane was excluded because of a nearby artery persistent in the image. (B) Example of A2 segmentation, where the algorithm could not separate the left and right A2 segments. If the previously published flow quantification algorithm could not quantify each separate A2 flow, then the individual artery was excluded from the analysis.

**Supplemental Table S1.** Baseline characteristics.

|  | Conventional ≥50% stenosis (n=68) | Near-occlusion (n=42) | P^a^ | Symptomatic near-occlusion (n=34) | Asymptomatic near-occlusion (n=8) | P^a^ | |
| --- | --- | --- | --- | --- | --- | --- | --- |
| Age (SD) | 74 (7) | 71 (6) | .02 | 72 (6) | 69 (7) | .21 | |
| Women, *n* (%) | 26 (38) | 9 (21) | .07 | 8 (24) | 1 (13) | .49 | |
| Systolic blood pressure (SD) | 150 (24) | 149 (22) | .69 | 150 (23) | 142 (18) | .38 | |
| Diastolic blood pressure (SD) | 76 (12) | 77 (10) | .92 | 76 (9) | 79 (11) | .43 | |
| Diabetes, *n* (%) | 20 (30) | 6 (14) | .07 | 4 (12) | 2 (25) | .34 | |
| Hypertension, n (%) | 58 (85) | 36 (86) | .95 | 29 (85) | 7 (88) | .87 | |
| Current smoker, *n* (%) | 10 (15) | 9 (21) | .37 | 7 (21) | 2 (25) | .78 | |
| Atrial fibrillation, *n* (%) | 10 (15) | 5 (12) | .68 | 4 (12) | 1 (13) | .95 | |
| Myocardial infarction, *n* (%) | 9 (13) | 5 (12) | .84 | 4 (12) | 1 (11) | .95 | |
| Current angina, *n* (%) | 1 (2) | 1 (2) | .73 | 1 (3) | 0 (0) | .62 | |
| Previous arterial revascularization, *n* (%) | 19 (28) | 10 (24) | .63 | 7 (21) | 3 (33) | .31 | |
| Presenting event: stroke, *n* (%) | 33 (58) | 12 (35) | <.001 | - | - | - | |
| Presenting event: TIA, *n* (%) | 20 (35) | 6 (18) |  | - | - |  | |
| Presenting event: retinal, *n* (%) | 4 (7) | 16 (47) |  | - | - |  | |
| Delay CTA–MRI (IQR) | 5 (3−7) | 4 (1−6) | .26 | 4 (2−7) | 1 (0−5) | .04 | |
| Stenosis diameter mm (SD)^b^ | 1.5 (0.5) | 0.5 (0.2) | <.001 | 0.6 (0.3) | 0.4 (0.2) | .04 | |
| Distal ICA diameter mm (SD) | 4.3 (0.7) | 2.6 (1.1) | <.001 | 2.8 (0.9) | 1.7 (1.4) | .01 | |
| ICA ratio (IQR)^c^ | 1.0 (0.9−1.1) | 0.7 (0.5−0.7) | <.001 | 0.7 (0.6−0.8) | 0.3 (0.1−0.6) | .01 | |
| ICA/ECA ratio (IQR) | 1.6 (1.4−1.8) | 1.1 (0.8−1.2) | <.001 | 1.1 (0.9−1.2) | 0.6 (0.1−1.1) | .04 | |
| Degree of conventional stenosis (SD)^d^ | 66 (11) | - | - | - | - | - | |
| Near-occlusion with full collapse, *n* (%) | - | 9 (21) | - | 5 (15) | 4 (50) | .03 | |
| SD: Standard deviation; IQR: inter-quartile range; TIA: transient ischemic attack; CTA: computed tomography; MRI: magnetic resonance imaging; ICA: internal carotid artery; ECA: external carotid artery; ICA ratio: ipsilateral/contralateral distal ICA diameter; ICA/ECA ratio: distal ipsilateral ICA/ipsilateral ECA; Stenosis diameter: smallest luminal stenosis diameter  ^a^ Two-sided independent sample t-test, Two-sided χ^2^ test, Mann-Whitney U test, where appropriate.  ^b^ 9 missing due to severe calcification (3 near-occlusion)  ^c^ 6 missing due to contralateral occlusion (1 near-occlusion)  ^d^ 6 missing due to severe calcification. Additionally, 2 included cases had <50% on CTA. | | | | | | |  |

**Supplemental Table S2.** Comparison of mean blood flow rates in ml/min (standard deviation) and median ratios (%, interquartile range) for cases with conventional ≥50% stenosis, unclear cause of small distal ICA, and near-occlusion.

| Artery | Conventional ≥50% stenosis (n=68) | Unclear cause of small distal ICA (n=17) | Near-occlusion (n=42) | P^a^ |
| --- | --- | --- | --- | --- |
| CBF_tot_ | 519 (88) | 489 (87) | 514 (88) | 0.47 |
| Hemi_tot_- ipsi ^b^ | 227 (55) | 208 (50) | 234 (51) | 0.27 |
| Hemi_tot_-contra ^b^ | 240 (64) | 234 (61) | 250 (60) | 0.65 |
| ICA-ipsi | 203 (73) | 124 (41) | 70 (45) | <0.001 |
| ICA-contra ^c^ | 209 (69) | 208 (98) | 267 (85) | 0.001 |
| M1-ipsi | 121 (28) | 100 (33) | 104 (38) | 0.01 |
| M1-contra | 125 (37) | 126 (35) | 128 (44) | 0.93 |
| A1-ipsi | 78 (59) | 30 (42) | -10 (43) | <0.001 |
| A1-contra | 64 (64) | 93 (59) | 133 (62) | <0.001 |
| A2-ipsi ^b^ | 54 (19) | 45 (22) | 57 (23) | 0.17 |
| A2-contra ^b^ | 59 (20) | 48 (24) | 55 (19) | 0.17 |
| BA | 122 (48) | 157 (84) | 183 (61) | <0.001 |
| P1-ipsi | 50 (34) | 73 (50) | 100 (40) | <0.001 |
| P1-contra | 47 (31) | 65 (56) | 67 (53) | 0.045 |
| P2-ipsi | 54 (22) | 67 (30) | 71 (28) | <0.001 |
| P2-contra | 58 (20) | 61 (27) | 68 (31) | 0.11 |
| Pcom-ipsi | 7 (22) | -1 (25) | -28 (33) | <0.001 |
| Pcom-contra | 12 (30) | 4 (33) | 8 (30) | 0.58 |
| Hemi_tot_-ipsi/contra ^b^ | 94 (87−105) | 90 (76−107) | 92 (84−100) | 0.54 |
| ICA-ipsi/ICA_sum_ | 46 (43−54) | 36 (26−50) | 20 (13−29) | <0.001 |
| ICA-ipsi/CBF_tot_ | 36 (32−42) | 23 (19−33) | 13 (8−18) | <0.001 |
| M1-ipsi/contra ^d^ | 95 (86−108) | 82 (66−100) | 82 (70−95) | <0.001 |
| A1-ipsi/A1_sum_ | 45 (31−62) | 25 (-4−58) | 0 (-41−10) | <0.001 |

Negative numbers indicate reversed flow. CBF_tot_ = ICA-ipsi + ICA-contra + BA; Hemi_tot_ = M1 + A2 + P2; ICA_sum_ = ICA-ipsi + ICA-contra; A1_sum_ = A1-ipsi + A1-contra.

^a^ One-way ANOVA for flow rates. Kruskal-Wallis test for flow ratios. Post-hoc test (P = .05) on flow rates in ICA-ipsi and A1-ipsi showed a difference between all three groups; flow rates in P1-ipsi and Pcom-ipsi were higher/reversed in near-occlusions than in conventional and uncertain cases.

^b^ 9 missing MRI due to the A2s being too close together to assess individually (3 unclear cause)

^c^ 6 missing due to contralateral occlusion (1 near-occlusion)

^d^ 1 missing due to bilateral MCA-occlusion on both CTA and MRI (near-occlusion)

**Supplemental Table S3.** Comparison of mean blood flow rates (ml/min, standard deviation) and median ratios (%, interquartile range) for cases with conventional stenosis <70% and ≥70%, and near-occlusion.

| Artery | Conventional <70% stenosis (n=36) | Conventional ≥70% stenosis (n=32) | Near-occlusion (n=42) | P^a^ |
| --- | --- | --- | --- | --- |
| CBF_tot_ | 515 (85) | 522 (91) | 514 (88) | 0.92 |
| Hemi_tot_- ipsi ^b^ | 232 (58) | 222 (52) | 234 (51) | 0.61 |
| Hemi_tot_-contra ^b^ | 241 (64) | 233 (66) | 250 (60) | 0.75 |
| ICA-ipsi | 222 (73) | 181 (68) | 70 (45) | <0.001 |
| ICA-contra ^c^ | 198 (65) | 220 (73) | 267 (85) | <0.001 |
| M1-ipsi | 125 (30) | 118 (27) | 104 (38) | 0.03 |
| M1-contra | 122 (35) | 129 (39) | 128 (44) | 0.76 |
| A1-ipsi | 94 (64) | 59 (47) | -10 (43) | <0.001 |
| A1-contra | 50 (67) | 80 (57) | 133 (62) | <0.001 |
| A2-ipsi ^b^ | 54 (20) | 54 (18) | 57 (23) | 0.77 |
| A2-contra ^b^ | 60 (21) | 58 (20) | 55 (19) | 0.59 |
| BA | 117 (46) | 128 (51) | 183 (61) | <0.001 |
| P1-ipsi | 46 (31) | 56 (37) | 100 (40) | <0.001 |
| P1-contra | 46 (31) | 49 (30) | 67 (53) | 0.05 |
| P2-ipsi | 54 (19) | 54 (25) | 71 (28) | 0.001 |
| P2-contra | 57 (22) | 58 (17) | 68 (31) | 0.12 |
| Pcom-ipsi | 12 (23) | 2 (21) | -28 (33) | <0.001 |
| Pcom-contra | 11 (30) | 14 (29) | 8 (30) | 0.73 |
| Hemi_tot_-ipsi/contra ^b^ | 95 (88−108) | 92 (80−104) | 92 (84−100) | 0.42 |
| ICA-ipsi/ICA_sum_ | 50 (46−61) | 45 (36−53) | 20 (13−29) | <0.001 |
| ICA-ipsi/CBF_tot_ | 39 (24−46) | 33 (27−40) | 13 (8−18) | <0.001 |
| M1-ipsi/contra ^d^ | 98 (92−114) | 90 (82−98) | 82 (70−95) | <0.001 |
| A1-ipsi/A1_sum_ | 49 (41−98) | 36 (21−57) | 0 (-41−10) | <0.001 |

^a^ One-way ANOVA for flow rates. Kruskal-Wallis test for flow ratios. Post-hoc test (P = .05) on flow rates in ICA-ipsi and A1-ipsi showed a difference between all three groups; flow rates in ICA-contra, A1-contra, BA, P1-ipsi, P2-ipsi and Pcom-ipsi higher/reversed in near-occlusion than in conventional stenosis; flow rates in M1-ipsi lower in near-occlusion than in conventional stenosis <70%.

^b^ 6 missing MRI due to the A2s being too close together to assess individually (2 near-occlusions)

^c^ 6 missing due to contralateral occlusion (1 near-occlusion)

^d^ 1 missing due to bilateral MCA-occlusion on both CTA and MRI (near-occlusion)

**Supplemental Table S4.** Comparison of mean blood flow rates in ml/min (standard deviation) and median ratios (%, interquartile range) for cases with near-occlusion with and without full collapse.

|  | Near-occlusion without full collapse (n=33) | Near-occlusion with full collapse (n=9) | P^a^ |
| --- | --- | --- | --- |
| CBF_tot_ | 527 (91) | 466 (60) | 0.07 |
| Hemi_tot_- ipsi ^b^ | 238 (54) | 222 (42) | 0.42 |
| Hemi_tot_-contra ^b^ | 245 (65) | 264 (35) | 0.41 |
| ICA-ipsi | 84 (40) | 21 (25) | <0.001 |
| ICA-contra ^c^ | 268 (82) | 264 (99) | 0.91 |
| M1-ipsi | 109 (35) | 94 (38) | 0.27 |
| M1-contra | 127 (46) | 132 (36) | 0.77 |
| A1-ipsi | -3 (42) | -36 (39) | 0.04 |
| A1-contra | 128 (62) | 151 (61) | 0.33 |
| A2-ipsi ^b^ | 58 (23) | 52 (25) | 0.47 |
| A2-contra ^b^ | 55 (19) | 57 (19) | 0.80 |
| BA | 184 (58) | 181 (75) | 0.90 |
| P1-ipsi | 102 (39) | 94 (42) | 0.61 |
| P1-contra | 67 (49) | 66 (68) | 0.98 |
| P2-ipsi | 71 (27) | 77 (32) | 0.58 |
| P2-contra | 66 (29) | 75 (38) | 0.41 |
| Pcom-ipsi | -30 (34) | -24 (32) | 0.63 |
| Pcom-contra | 6 (28) | 15 (38) | 0.47 |
| Hemi_tot_-ipsi/contra ^b^ | 93 (85−102) | 91 (72−96) | 0.15 |
| ICA-ipsi/ICA_sum_ | 22 (16−30) | 4 (2−15) | 0.001 |
| ICA-ipsi/CBF_tot_ | 15 (11−19) | 1 (1−9) | <0.001 |
| M1-ipsi/contra ^d^ | 82 (72−96) | 71 (57−86) | 0.19 |
| A1-ipsi/A1_sum_ | 0 (-18−19) | -27 (-59−0) | 0.045 |
| Negative numbers indicate reversed flow. CBF_tot_ = ICA-ipsi + ICA-contra + BA; Hemi_tot_ = M1 + A2 + P2; ICA_sum_ = ICA-ipsi + ICA-contra; A1_sum_ = A1-ipsi + A1-contra.  ^a^ Two-sided independent sample t-test for flow ratios. Mann-Whitney U-test for flow ratios.  ^b^ 2 missing MRI due to the A2s being too close together to assess individually (without full collapse)  ^c^ 1 missing data due to contralateral occlusion  ^d^ 1 missing due to bilateral MCA-occlusion on both CTA and MRI (near-occlusion) | | | |
